# Metastatic Pulmonary Calcification Detected on ^18^F-FDG PET/CT and ^99m^Tc-MDP Bone Scan

**DOI:** 10.3390/diagnostics11091627

**Published:** 2021-09-06

**Authors:** Miju Cheon, Jang Yoo

**Affiliations:** Department of Nuclear Medicine, Veterans Health Service Medical Center, Seoul 05368, Korea; jang8214.yoo@gmail.com

**Keywords:** metastatic calcification, FDG, PET/CT, bone scan

## Abstract

Metastatic calcification relates to abnormal calcification resulting from hypercalcemia and can affect soft tissues, skeletal muscle, myocardium, lungs, stomach, kidneys, and blood vessels. We describe a case of metastatic pulmonary calcification in a 71-year-old male, images with ^18^F-fluorodeoxyglucose (FDG) PET/CT and ^99m^Tc- methylene diphosphonate (MDP) bone scan.

A 71-year-old Asian man, ethnically Korean, had a long history of chronic kidney disease and prostate cancer (BMI 21.9 kg/m^2^). He was on diuretic therapy for chronic kidney disease for 4 years, followed by oral adsorptive carbon therapy for 13 months. He had been on hormonal therapy for prostate cancer (cT2cN0M0) for 9 months. He was hospitalized elsewhere for poor oral intake and general weakness. Laboratory studies at that time revealed the following (reference ranges provided parenthetically): calcium 15.5 mg/dL (8.2–10.2 mg/dL); phosphorus 7.8 mg/dL (2.5–4.5 mg/dL); creatinine 3.67 mg/dL (0.7–1.2 mg/dL); total 25-hydroxyvitamin D level 12.06 ng/mL (approx. 30 ng/mL); and parathyroid hormone 15.71 pg/mL (15–65 pg/mL). ^18^F-fluorodeoxyglucose (FDG) PET/CT was performed to exclude the possibility of malignancy-related hypercalcemia. ^18^F-FDG) PET/CT images were acquired 1 h after intravenous injection of 238 MBq of ^18^F-FDG. The PET/CT images showed an increase in FDG uptake in the bilateral lower lungs (Figure 1). There was no focal FDG uptake suggesting malignancy. Correlative non-contrast CT images of the thorax revealed diffuse, hazy ground-glass opacities in the bilateral lower lungs. These PET/CT findings are nonspecific and can be seen in patients with diverse diseases such as atypical bacterial and viral infections, alveolar hemorrhage, pulmonary edema, diffuse alveolar damage, pulmonary embolism, chemotherapy-induced pneumonitis, acute respiratory distress syndrome, or interstitial lung disease [1,2,3,4,5,6,7]. Chest radiography (Figure 2) showed only peribronchial infiltration at both lower lungs. However, since other differential diagnoses were inappropriate for the patient’s clinical condition and a patient with chronic kidney disease accompanied by hypercalcemia, metastatic pulmonary calcification was considered one differential diagnosis.

Further examination with a bone scan was therefore recommended. Since the bone scan is a sensitive test for diagnosing metastatic pulmonary calcification, we performed a bone scan to identify metastatic pulmonary calcification and determine whether bone metastasis existed [8]. The patient subsequently underwent a ^99m^Tc-methylene diphosphonate (MDP) bone scan. It also revealed significantly increased diffuse uptake in the bilateral lower lung fields (Figure 3). The scan was negative for osteoblastic skeletal metastasis. These findings were suggestive of metastatic calcification. Both exams indicated the lesion to be caused by metastatic pulmonary calcification. The patient was treated with hemodialysis, and his follow-up data is not available because he transferred to another hospital.

Metastatic pulmonary calcification is a frequently underdiagnosed disease. Because usual imaging modalities such as chest radiographs and CT scan findings are not specific [8,9]. Only a few reports are available demonstrating the ability of ^18^F-FDG PET/CT to detect metastatic pulmonary calcification [10,11]. However, no report presented both bone scan and ^18^F-FDG PET/CT findings in patients with metastatic pulmonary calcification. In conclusion, in patients with chronic kidney disease, when hypercalcemia is present and PET/CT shows ground-glass opacity with mild FDG uptake, metastatic pulmonary calcification can be considered one of the differential diagnoses, though this is rare.

## Figures and Tables

**Figure 1:**
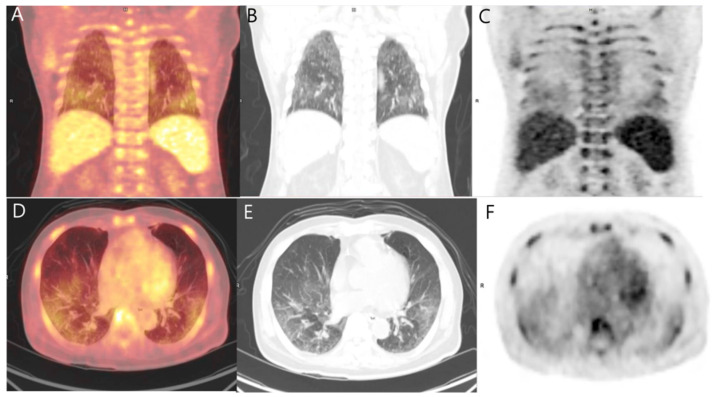
^18^F-FDG PET/CT coronal (**A**–**C**) and axial (**D**–**F**) images of the thorax demonstrated bilateral high-grade uptake in the posterior and inferior aspect of the lungs and diffuse low-grade uptake in the remainder of the lungs. Non-contrast CT image of PET/CT (**B**,**E**) revealed diffuse, hazy ground-glass opacities in the bilateral lower lungs.

**Figure 2:**
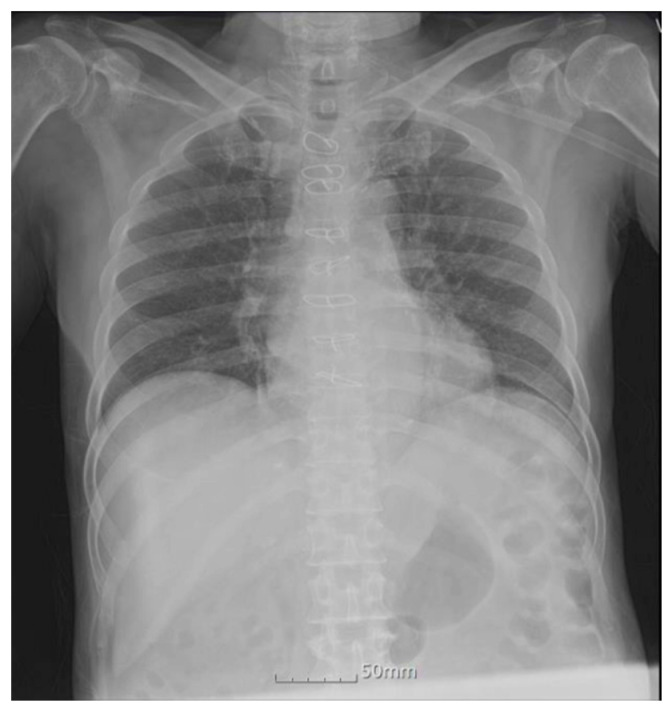
Chest radiography showed non-specific infiltrations at both lower lungs.

**Figure 3:**
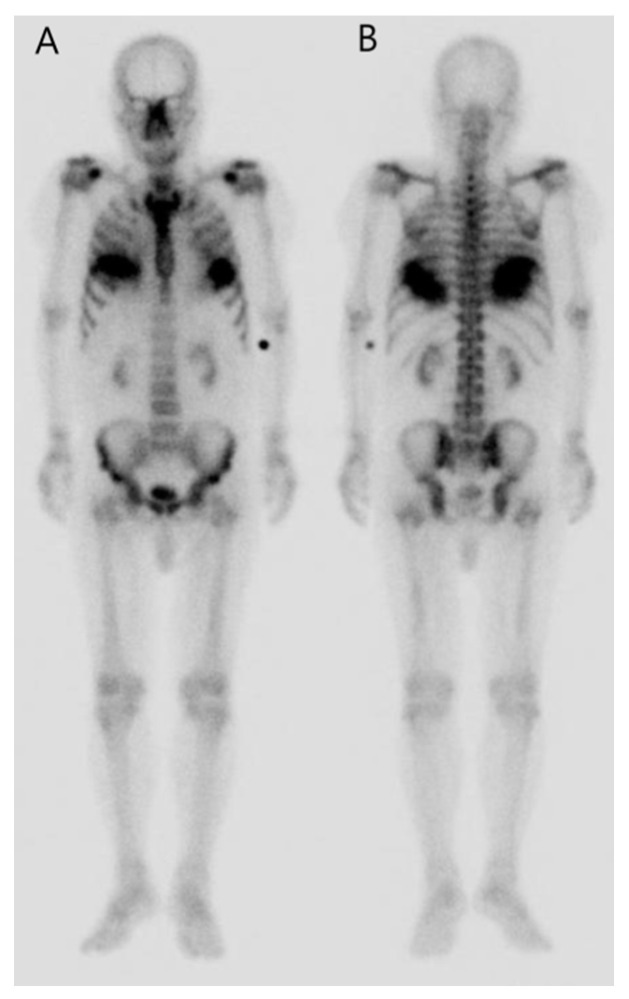
Both anterior (**A**) and posterior (**B**) images of the bone scan revealed significantly increased diffuse MDP uptake in the bilateral lower lung fields.

## Data Availability

The data that support the findings of this study are available from the corresponding author M.C., upon reasonable request.
